# Terahertz Emission Spectroscopy of Ultrafast Coupled Spin and Charge Dynamics in Nanometer Ferromagnetic Heterostructures

**DOI:** 10.3390/nano12234267

**Published:** 2022-11-30

**Authors:** Zhangshun Li, Yexin Jiang, Zuanming Jin, Zhuoyi Li, Xianyang Lu, Zhijiang Ye, Jin-Yi Pang, Yongbing Xu, Yan Peng

**Affiliations:** 1 Terahertz Technology Innovation Research Institute, Terahertz Spectrum and Imaging Technology Cooperative Innovation Center, Shanghai Key Lab of Modern Optical System, University of Shanghai for Science and Technology, Shanghai 200093, China; 2 Jiangsu Provincial Key Laboratory of Advanced Photonic and Electronic Materials, School of Electronic Science and Engineering, Nanjing University, Nanjing 210093, China

**Keywords:** spintronic THz emitters, ultrafast demagnetization, spin-to-charge current conversion, THz emission spectroscopy

## Abstract

Due to its high sensitivity and because it does not rely on the magneto-optical response, terahertz (THz) emission spectroscopy has been used as a powerful time-resolved tool for investigating ultrafast demagnetization and spin current dynamics in nanometer-thick ferromagnetic (FM)/heavy metal (HM) heterostructures. Here, by changing the order of the conductive HM coating on the FM nanometer film, the dominant electric dipole contribution to the laser-induced THz radiation can be unraveled from the ultrafast magnetic dipole. Furthermore, to take charge equilibration into account, we separate the femtosecond laser-induced spin-to-charge converted current and the instantaneous discharging current within the illuminated area. The THz emission spectroscopy gives us direct information into the coupled spin and charge dynamics during the first moments of the light–matter interaction. Our results also open up new perspectives to manipulate and optimize the ultrafast charge current for promising high-performance and broadband THz radiation.

## 1. Introduction

Laser-induced ultrafast magnetization phenomena in materials attracts strong interest from both basic knowledge and technology perspectives [1,2,3,4,5]. The ability to efficiently control the spin of ferromagnets on a femtosecond (fs) timescale paves the way to ultrafast optical writing of magnetic memory and data processing [6,7,8,9,10]. Although many experiments and theories have been presented lately, the clear physical mechanism of the processes occurring during the interaction of ultrashort laser pulses with the spin order remains largely unclear, even though understanding dynamics on this timescale is critical for both physical and practical insights into the spin diffusion, precession, relaxation, and spin–orbit interaction [11,12,13,14,15].

The key experimental techniques routinely employed so far are the time-resolved magneto-optical Kerr effect (TR-MOKE) [16,17] or soft X-ray pulses [18]. These techniques have provided invaluable information concerning the transient evolution of magnetization in magnetic materials on fs and longer timescales. However, both of these techniques, as based on the pump–probe cross-correlation, are inherently ambiguous towards the material response during the interaction of the laser pulse with the material. This is due to the zero-time ambiguity of the pump-probe measurement and the possible occurrence of coherent effects or artifacts between pump and probe pulses [5]. As the propagation of terahertz (THz) waves in materials is mainly affected by the motion of the electrons, it provides the most direct way to probe the magneto-transport properties on an ultrafast time scale. Over the past years, THz time-domain spectroscopy (THz-TDS) has provided the opportunity for not only investigating biomedical systems [19,20], but also uncovering the spin dynamics in both antiferromagnetic [21,22,23,24,25] and ferromagnetic materials [26,27,28,29,30]. Furthermore, in contrast to the TR-MOKE method, THz emission spectroscopy does not rely on the magneto-optical response and has been used as a fingerprint identification of the ultrafast demagnetization [31,32], inverse spin Hall effect (ISHE) [33,34,35], inverse spin–orbit torque [36], inverse Rashba–Edelstein effect (IREE) [37,38,39], and anomalous Hall effect (AHE) [40]. In addition, THz emission spectroscopy has the capability of small magnetic field change detection in the μT range [41] and near-field imaging with subwavelength resolution [42].

It is widely understood that the ultrafast response of a laser-excited pure ferromagnetic system is dominated by the ultrafast demagnetization (UDM) [1,43]. In a ferromagnetic (FM) thin film, the excess energy of laser-excited hot electrons is transferred to spin and lattice subsystems, leading to the local magnetic dipole THz radiation, $\;E\left(t\right)\propto \frac{\partial^{2}M}{\partial t^{2}}$ [31,32,44], where ***M*** is the magnetization of the FM layer. On the other hand, the spin-to-charge current conversion (SCC) via ISHE, IREE, or AHE converts the spin current ($j_{s}$) into a charge current ($j_{c}$) via spin–orbit coupling, $\;j_{c}\propto j_{s}\times \frac{M}{\left|M\right|}$. Such a response acts as an electronic dipole THz radiation, $\;E\left(t\right)\propto \frac{\partial j_{c}}{\partial t}$ [45,46,47]. Remarkably, the working principle of the THz radiation remains entangled with the microscopic origin on how an ultrafast laser pulse modifies the magnetic system after fs laser excitation. In addition, the far field detection of THz emission does not give access to the exact current distribution in the emitter. To disentangle the physical origins is also crucial to maximize the generation efficiency of THz pulses.

In this work, we use time-resolved THz emission spectroscopy to study the spin and charge dynamics in laser-excited MgO (substrate)//Pt/CoFeB/Ta and MgO//Ta/CoFeB/Pt heterostructures. Such a choice of samples allowed us to accurately trace the measured THz emission back to the transients of UDM and SCC contributions at any moment during and after the interaction of the fs laser pulse with the sample. As a result, we gain direct access to the zero-time response of the laser-excited transient spin voltage dynamics in ferromagnetic/heavy metal (FM/HM) heterostructures, which is knowledge difficult to obtain by any other methods. Our work demonstrates potential for an ultrafast THz magnetometer and spintronic THz emitter.

## 2. Materials and Methods

### 2.1. Thin Films Preparation

Two types of samples were prepared. Here, 6 nm-thick Pt(2)/CoFeB(2)/Ta(2) and Ta(2)/CoFeB(2)/Pt(2) heterostructure films were grown on single-crystalline MgO (100) substrates. For the Pt/CoFeB/Ta sample, the 2 nm-thick Pt layer was firstly deposited by magnetron sputtering on a MgO substrate. Then, a 2 nm-thick CoFeB layer was deposited on the Pt layer, and a 2 nm-thick Ta layer was deposited on the CoFeB. The deposition rate of the Pt (the DC power was 10 W) layer, the CoFeB (the DC power was 10 W) layer, and the Ta (the DC power was 10 W) layer were 0.3, 0.15, and 0.3 Å/s at $7\times 10^{-3}$ Torr, respectively. The samples were annealed in situ after deposition at 250 °C for 60 min. For the Ta/CoFeB/Pt sample, the deposition process is similar under identical preparation conditions.

### 2.2. Terahertz Emission Spectroscopy

**Figure 1:**
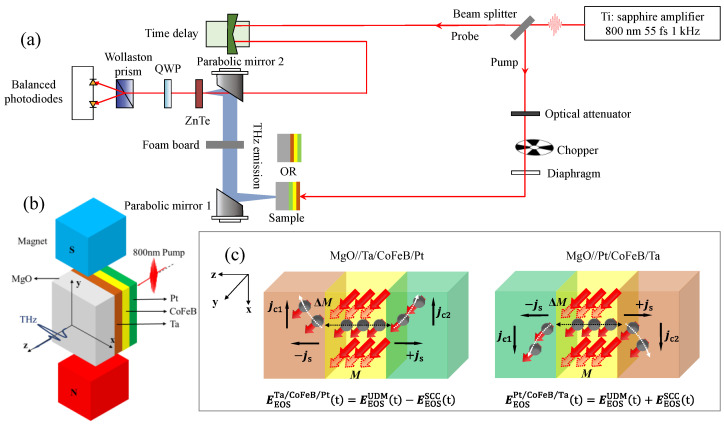
Generation of THz radiation from trilayer MgO//Pt/CoFeB/Ta and MgO//Ta/CoFeB/Pt stacks. (**a**) Schematic geometry for the THz emission spectroscopy in the transmission configuration. (**b**) The trilayer structure is excited by an 800 nm fs pump pulse and applied in-plane magnetic field of ±200 mT. (**c**) Physical origins of SCC- and UDM-based THz generations. The black circles with arrows show the electrons with spins. The light red arrow and solid red arrow represent the magnetization ***M*** of the CoFeB layer, before and after ultrafast demagnetization, respectively. The black dotted arrows represent the diffusion of spin currents toward the Ta or Pt layer. The white dotted arrows represent the deflection and conversion of spin currents ±***j***_s_ into charge currents ***j***_c_ in both the Pt and Ta layers.

In Figure 1a,b, the conceptual schematic of our THz emission spectroscopy is shown. The sample is nearly homogeneously pumped by a normal incident of 800 nm (photon energy of 1.55 eV), pulse duration of 55 fs, and a repetition rate of a 1 kHz optical pulse. The beam is split into a pump beam and a probe beam for THz detection. The pump beam is angled at a normal incident onto the samples. The magnetization of the sample was set by an applied external magnetic field parallel to the *y*-axis (in-plane). The amount of externally applied magnetic field was kept ~200 mT (sufficient to saturate the CoFeB layer). As the heterostructure is thinner than the laser penetration depth (~20 nm) [48], the excitation of hot electrons can be assumed to be homogeneous across the thickness of the film. We analyze the THz emissions from both SCC and UDM contributions by measuring the far-field free-space electro-optic sampling (EOS). The linearly-polarized THz emission was collected by a parabolic mirror and detected by a 1 mm-thick <110> ZnTe crystal gated by 55 fs, 800 nm laser pulses, using two parabolic mirrors. A chopper modulates the pump laser beam with a frequency of 0.5 kHz. The emitted THz pulses are sampled by delaying the probe laser beam time, and the amplified signal is recorded by a lock-in amplifier. All the measurements were performed at room temperature, and the spectrometer was purged with dry air to avoid the THz absorption by atmospheric water.

Figure 1c shows that the excitation induces a transient spin voltage that launches spin currents flowing from CoFeB to Ta and from CoFeB to Pt. The trilayer emitter converts the bidirectional spin currents (−***j***_s_ and +***j***_s_) flowing into the lateral charge currents ***j***_c_ within Ta and Pt, due to the ISHE. The time-varying ***j***_c_ perpendicular to the ***j***_s_ (scales with the ***M*** of CoFeB) and the direction of ***M*** gives rise to the emission of THz transients. We chose Ta and Pt because their signs of the spin Hall angle are opposite and, thus, their THz emissions add up [49]. Since the thickness of the used materials is small (nanometers), the THz radiations originating from the two HM layers have a negligible phase difference. In addition, a sizable contribution coming from the time-dependent UDM is also expected [31,32,50].

## 3. Results and Discussion

**Figure 2:**
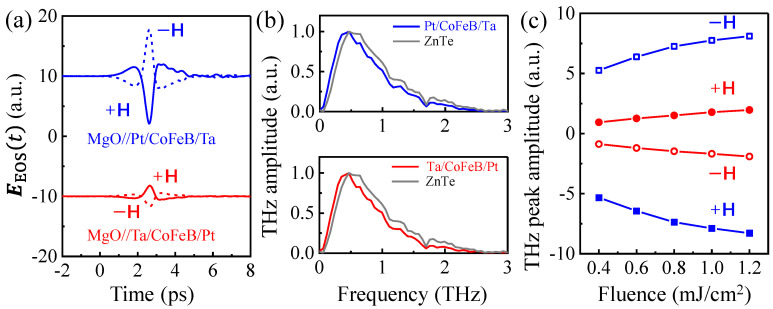
Terahertz waveforms at the different excitation geometries. (**a**) THz EOS waveforms $E_{\mathrm{EOS}}\left(t\right)$ of the photoexcited MgO//Pt/CoFeB/Ta and MgO//Ta/CoFeB/Pt stacks. The zero-time delay corresponds to the arbitrary starting point. (**b**) The corresponding normalized Fourier transform of (**a**). The light-gray curve is the THz emission from a ZnTe crystal. (**c**) The pump fluence-dependence of THz peak amplitude from two samples at ±***H***. Damage is not observed in the samples at the maximum pump fluence used here.

According to the experimental scheme shown in Figure 1, Figure 2a shows the typical time-domain THz EOS signals, $E_{\mathrm{EOS}}\left(t\right)$, generated from both Pt/CoFeB/Ta and Ta/CoFeB/Pt stacks at a pump fluence of 1.0 mJ/cm^2^. Both THz transients are inverted symmetrically, as the direction of ***M*** is reversed with an external magnetic field of ±200 mT. The polarization of the emitted THz pulses for both samples follows the direction perpendicular to ***M*** (along the *y*-axis), which indicates a strong connection between the THz emission and the magnetic order. We have also measured the THz emission of a 0.5 mm-thick ZnTe reference emitter under the same excitation condition. As shown in Figure 2b, the normalized Fourier spectra $\left|E_{\mathrm{EOS}}\left(\omega \right)\right|$ versus frequency ω/2π of ZnTe covers a slightly higher range of frequencies, as compared with the trilayer stacks.

Figure 2a shows that the polarity of THz emissions in the Pt/CoFeB/Ta is found to reverse its sign, compared with the Ta/CoFeB/Pt, under the same experimental conditions. Given that the optical absorbance does not depend on the order of the sample, thus, a direction-dependent mechanism explains such polarity reversal of the THz emitted field. As shown in Figure 1c, by reversing the order of the Ta and Pt layer, the SCC model predicts a polarity reversal of the THz radiation owing to a reversion of charge currents within bilateral HM layers. Figure 2c shows that the THz peak amplitude increases with increased pump fluence and then reaches saturation. More THz waveforms at different pump fluences can be found in the Supplementary Materials.

It is also noted that the absolute value of THz signal from Pt/CoFeB/Ta is larger than that from Ta/CoFeB/Pt. That means the far-field $E_{\mathrm{EOS}}\left(t\right)$ signals for two samples include both SCC and UDM contributions. Under the simplified assumption that the polarity of the THz emission by SCC is opposite for two samples, the UDM contribution does not change under the fixed magnetic field. Thus, $E_{\mathrm{EOS}}^{\mathrm{Pt}/\mathrm{CoFeB}/\mathrm{Ta}}\left(t\right)=E_{\mathrm{EOS}}^{\mathrm{UDM}}\left(t\right)+E_{\mathrm{EOS}}^{\mathrm{SCC}}\left(t\right)$ and $E_{\mathrm{EOS}}^{\mathrm{Ta}/\mathrm{CoFeB}/\mathrm{Pt}}\left(t\right)=E_{\mathrm{EOS}}^{\mathrm{UDM}}\left(t\right)-E_{\mathrm{EOS}}^{\mathrm{SCC}}\left(t\right)$. Therefore, the distinct contributions of UDM- and SCC-based THz emission can be expressed as follows:

(1) $$E_{\mathrm{EOS}}^{\mathrm{UDM}}\left(t\right)=\left(E_{\mathrm{EOS}}^{\mathrm{Pt}/\mathrm{CoFeB}/\mathrm{Ta}}\left(t\right)+E_{\mathrm{EOS}}^{\mathrm{Ta}/\mathrm{CoFeB}/\mathrm{Pt}}\left(t\right)\right)/2$$

(2) $$E_{\mathrm{EOS}}^{\mathrm{SCC}}\left(t\right)=\left(E_{\mathrm{EOS}}^{\mathrm{Pt}/\mathrm{CoFeB}/\mathrm{Ta}}\left(t\right)-E_{\mathrm{EOS}}^{\mathrm{Ta}/\mathrm{CoFeB}/\mathrm{Pt}}\left(t\right)\right)/2$$

**Figure 3:**
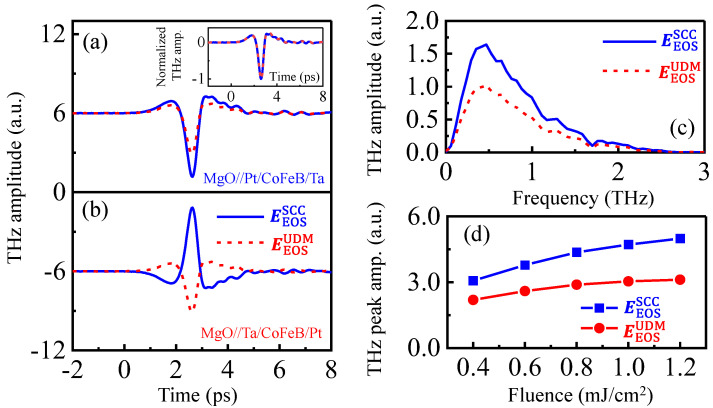
The $E_{\mathrm{EOS}}\left(t\right)$ is decomposed into SCC and UDM contributions. The temporal $E_{\mathrm{EOS}}^{\mathrm{SCC}}\left(t\right)$ and $E_{\mathrm{EOS}}^{\mathrm{UDM}}\left(t\right)$ obtained in (**a**) Pt/CoFeB/Ta and (**b**) Ta/CoFeB/Pt, with a fluence of 1.0 mJ/cm^2^. Inset shows the normalized $E_{\mathrm{EOS}}^{\mathrm{SCC}}\left(t\right)$ and $E_{\mathrm{EOS}}^{\mathrm{UDM}}\left(t\right)$. (**c**) The corresponding Fourier transforms of $E_{\mathrm{EOS}}^{\mathrm{SCC}}\left(t\right)$ and $E_{\mathrm{EOS}}^{\mathrm{UDM}}\left(t\right)$ of (**a**). (**d**) The THz peak amplitudes of SCC and UDM contributions versus pump fluence.

Figure 3a,b plots the $E_{\mathrm{EOS}}^{\mathrm{UDM}}\left(t\right)$ (red) and $E_{\mathrm{EOS}}^{\mathrm{SCC}}\left(t\right)$ (blue) in the Pt/CoFeB/Ta and Ta/CoFeB/Pt samples. The THz peak amplitude by $E_{\mathrm{EOS}}^{\mathrm{UDM}}\left(t\right)$ reaches around 37% in magnitude compared with $E_{\mathrm{EOS}}^{\mathrm{SCC}}\left(t\right)$, which is consistent with the result reported by Liu et al. [51]. The most remarkable finding is that THz emission efficiency of a trilayer stack can be improved by optimizing the order of the Pt and Ta layers. Note also that the normalized UDM- and SCC-based THz emissions exhibit similar temporal waveforms on the picosecond time scales (inset of Figure 3a) and spectrum on the frequency domain (Figure 3c). In Figure 3d, both UDM and SCC contributions are shown as functions of pump fluences from 0.4 to 1.2 mJ/cm^2^. Both SCC and UDM increase with the pump fluence, indicating that (1) hot electron transport is not hindered by the interface between CoFeB and HM layers, and (2) UDM-based THz emission is proportional to the magnitude of ultrafast demagnetization, before optical damage. All the experimental observations are consistent with the result reported by Rouzegar et al., and strongly demonstrate that SCC and UDM are driven by the same force, the transient spin voltage [52,53,54], $\dot{M}\propto j_{s}\propto \mu^{↑}-\mu^{↓}$, where $\mu^{↑}$ and $\mu^{↓}$ are the chemical potentials of majority- and minority-spin electrons in the FM layer, respectively.

**Figure 4:**
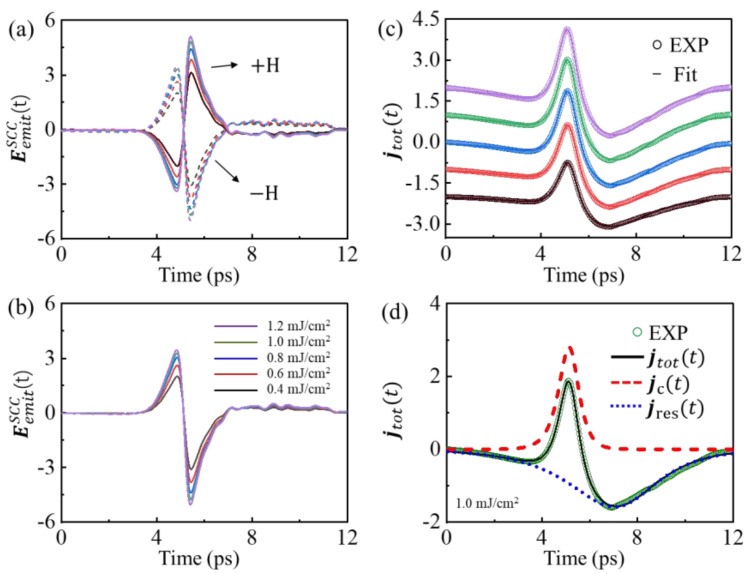
(**a**) The near-fields of the emitted THz radiation $E_{\mathrm{EOS}}^{\mathrm{SCC}}\left(t\right)$ for opposite applied magnetic fields with different pump fluences. (**b**) The difference $E_{emit}^{\mathrm{SCC}}\left(t\right)=\frac{E_{\mathrm{EOS}}^{\mathrm{SCC}}\left(t,+M\right)-E_{\mathrm{EOS}}^{\mathrm{SCC}}\left(t,-M\right)}{2}\;$ odd in ***M***. (**c**) The temporal profiles for charge current $j_{tot}\left(t\right)$ (vertical offset for clarity). The solid lines are the fits of Equation (4). (**d**) The complete charge current dynamics ***j****_tot_*(*t*) comprise both photoinduced SCC current ***j****_c_*(*t*) and restoration current ***j****_res_*(*t*), as shown by the red dashed and blue dotted line, respectively.

Note that the measured $E_{\mathrm{EOS}}\left(\omega \right)$ are the convolution of the transient THz field at the emitter $E_{emit}\;\left(\omega \right)$ with the response function of our measurement setup $H_{spectr}\left(\omega \right)$ [55,56], $E_{\mathrm{EOS}}\left(\omega \right)=E_{emit}\;\left(\omega \right)\times H_{spectr}\left(\omega \right)$, where $H_{spectr}\left(\omega \right)$ accounts for the propagation function of the THz pulse from the emitter to the EOS detector (see Supplementary Materials for details). Thus, we further calculate the near-fields of the emitted THz radiation $\;E_{emit}^{\mathrm{SCC}}\left(t,\pm M\right)$ from the measured $E_{\mathrm{EOS}}^{\mathrm{SCC}}\left(t,\pm M\right)$, as shown in Figure 4a. As we are only interested in odd effects in the sample magnetization, we, thus, focus on the signal $E_{emit}^{\mathrm{SCC}}\left(t\right)=\frac{E_{\mathrm{EOS}}^{\mathrm{SCC}}\left(t,+M\right)-E_{\mathrm{EOS}}^{\mathrm{SCC}}\left(t,-M\right)}{2}$, as shown in Figure 4b, which minimizes the artifacts of non-magnetic origin.

According to the charge conservation, the photoinduced SCC current ***j****_c_* works as a current source, which charges a capacity within the illuminated area. The system is transiently charged locally. Then, the capacity will be discharged, which leads to a restoration of charge neutrality current ***j****_res_*, which is dependent on the resistance of the surrounding material [57]. Therefore, the dynamics of total charge current is as follows:

(3) $$j_{\mathrm{tot}}\left(t\right)=j_{c}\left(t\right)+j_{res}\left(t\right)$$

The preceding equation describes a restoration of a charged state to a charge neutrality state. Figure 4c shows the measured total charge current transients ***j****_tot_*$\left(t\right)$, which is proportional to the time integration of the THz near-fields in Figure 4b. The ***j****_tot_*$\left(t\right)$ pulse consists of a positive peak followed by a negative one, which changes sign around 1.4 ps after onset. To gain more insight into our data, ***j****_tot_*$\left(t\right)$ is fitted by a current profile [58,59], as follows:

(4) $$j_{tot}\left(t\right)=\underset{i=c,\;res}{\sum }A_{i}\frac{e^{\alpha_{i}}}{e^{\alpha_{i}}+1}\times e^{\beta_{i}}$$

where $\alpha_{i}=\frac{\left(t-t_{0,i}\right)-\tau_{rise,\;i}/2}{\tau_{rise,i}/4},\;\beta_{i}=-\frac{t-t_{0,i}}{\tau_{decay,i}}$. Here, $\tau_{rise,i}$ and $\tau_{decay,i}$ are the rise and decay time constants of ***j****_i=c,res_*, respectively. Furthermore, $A_{i}$ and *t*_0,_*_i_* are the amplitude and time at peak of the current profile, respectively. The solid curves show the fitting results for different pump fluences. To aid comparison, the individual ***j**_c_*(*t*) (red dashed curve) and ***j****_res_*(*t*) (blue dotted curve) are separated, as shown in Figure 4d. In the beginning, the system response ***j***_tot_(*t*) rises rapidly with the slope of ***j****_c_*(*t*). After a very short time, the ***j***_tot_(*t*) decreases and change the sign, due to a negative contribution of ***j****_res_*(*t*). This restoration current is asymmetric around its peak, and the peak of ***j****_res_*(*t*) is slightly delayed with respect to the peak of ***j****_c_*(*t*). More figures of individual current dynamics at different pump fluences can be seen in the Supplementary Materials.

**Figure 5:**
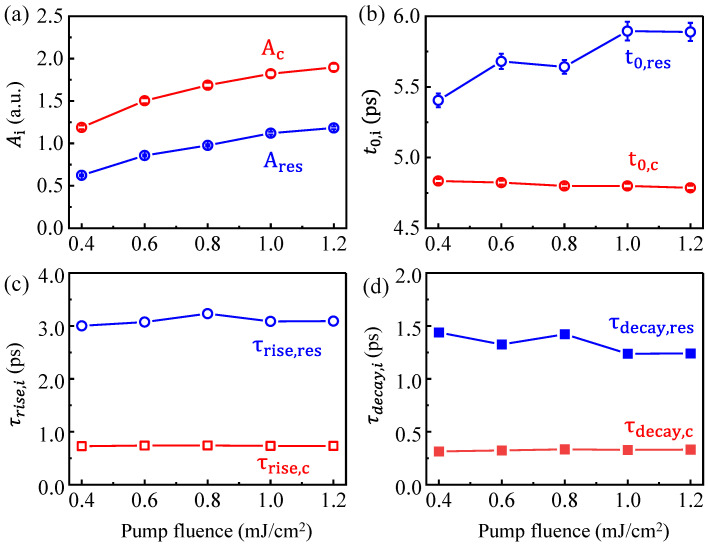
Pump fluence evolutions of (**a**) the amplitude and (**b**) time at peak of the ***j****_c_*(*t*) and ***j****_res_*(*t*) current profiles. Pump fluence-dependent (**c**) rise and (**d**) decay time constants of ***j****_c_*(*t*) and ***j****_res_*(*t*), respectively.

Figure 5 summarizes the extracted $A_{i}$, *t*_0,*i*_, $\tau_{rise,i}$, and $\tau_{decay,i}$ of two current pulses versus the pump fluence. Figure 5a shows that the larger the pump fluence, the higher the amplitude of the currents of ***j****_c_*(*t*) and ***j***_res_(*t*). This is a trivial consequence of the fact that due to the linear relationship between ***j****_c_* and ***j***_s_, the increase in amplitude results from an increased excited spin current density upon deposition of the pump-pulse energy. Importantly, we observe the different time positions of ***j****_c_(t*) and ***j****_res_*(*t*) with different pump fluences. The *t*_0,*c*_ for ***j****_c_*(*t*) is observed as being pump fluence-independent. On the other hand, the *t*_0,*res*_ of ***j****_res_*(*t*) is slightly increased with the increase in pump fluence (Figure 5b). Figure 5 c,d shows that the rise and decay time constants of the charge currents are nearly pump fluence-independent. Here, $\tau_{rise}=0.74\pm 0.002\;$ ps and $\tau_{decay}=0.33\pm 0.005$ ps of ***j****_c_*(*t*) correspond to the timescale of transient spin currents, which are much faster than the 3.10 ± 0.005 ps and 1.33 ± 0.090 ps of ***j****_res_*(*t*). The rise and decay of the ***j****_res_*(*t*) mainly depends on the conductivity of the heterostructure. Therefore, a more proper consideration of the complex interplay between spin voltage profile, spin-to-charge conversion, and material properties is required to maximize and shape the THz emissions [60], which will be our future research focus.

## 4. Conclusions

In summary, we have shown that high-performance broadband THz emission arises from both the magnetic dipolar radiation due to the ultrafast demagnetization and transient current radiation due to the spin–charge conversion. We find that the UDM contribution has the same time evolution as the SCC one, suggesting that they are driven by the same force, namely a generalized spin voltage. Finally, by taking the charge equilibration into account, we found that the THz generation depends on the overall superimposed fs laser-induced spin-to-charge converted current and an instantaneous backflow current. Our analysis distinguishes the contributions from the SCC and backflow charge current, which is essential to the underlying spin dynamics and optimization of spintronic THz emitters.

## Data Availability

The data of this study are available in the article, Supplementary Materials and upon request to the authors.

## Supplementary Materials

The following supporting information can be downloaded at: https://www.mdpi.com/article/10.3390/nano12234267/s1, Figure S1: (a) THz EOS waveforms $E_{\mathrm{EOS}}\left(t\right)$ at different pump fluences for two samples. (b) and (c) are the Fourier transform of Pt/CoFeB/Ta and Ta/CoFeB/Pt in (a), respectively. Solid lines and dotted lines are the data measured with +H and −H, respectively; Figure S2: The fittings of charge currents with ***j****_c_*(*t*) and ***j****_res_*(*t*) contributions at difference pump fluence range from 0.4 mJ/cm^2^ to 1.2 mJ/cm^2^.
