# Supplementary material for: Terahertz Emission Spectroscopy of Ultrafast Coupled Spin and Charge Dynamics in Nanometer Ferromagnetic Heterostructures
Source: Nanomaterials (Basel). 2022 Nov 30;12(23):4267. doi: 10.3390/nano12234267 (PMC9741209; doi:10.3390/nano12234267)
Supplement: Supplementary file 1 [file nanomaterials-12-04267-s001.zip › nanomaterials-2042119-supplementary.pdf]

nanomaterials

Supplementary Materials

Terahertz Emission Spectroscopy of Ultrafast Coupled Spin and Charge Dynamics in Nanometer Ferromagnetic Heterostructures

Zhangshun Li [1,†], Yexin Jiang [1,†], Zuanming Jin [1,*], Zhuoyi Li [2], Xianyang Lu [2,*], Zhijiang Ye [1], Jin-Yi Pang [1], Yongbing Xu [2] and Yan Peng [1,*]

[1] Terahertz Technology Innovation Research Institute, Terahertz Spectrum and Imaging Technology Cooperative Innovation Center, Shanghai Key Lab of Modern Optical System, University of Shanghai for Science and Technology, Shanghai 200093, China
[2] Jiangsu Provincial Key Laboratory of Advanced Photonic and Electronic Materials, School of Electronic Science and Engineering, Nanjing University, Nanjing 210093, China
* Correspondence: physics_jzm@usst.edu.cn (Z.J.); xylu@nju.edu.cn (X.L.); py@usst.edu.cn (Y.P.)
† These authors contributed equally to this work.

1. THz waveforms at different pump fluences

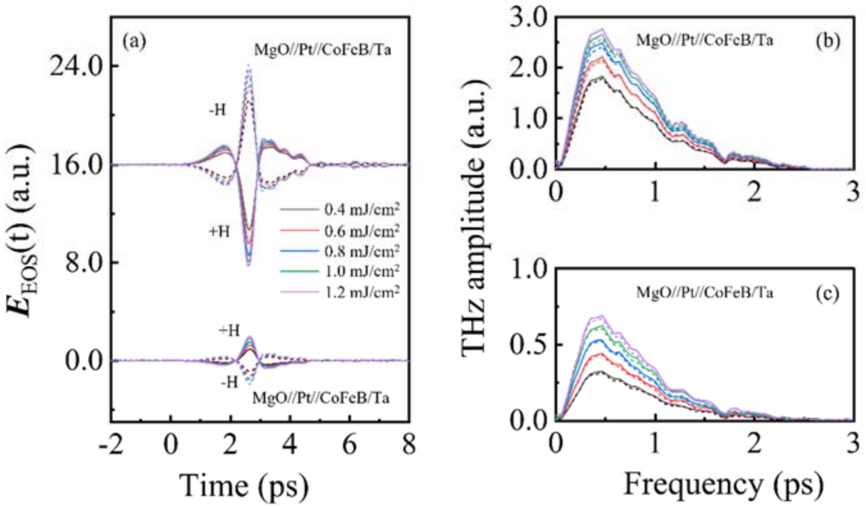

Figure S1. (**a**) THz EOS waveforms $\boldsymbol{E}_{\mathrm{EOS}}(t)$ at different pump fluences for two samples. (**b**) and (**c**) are the Fourier transform of Pt/CoFeB/Ta and Ta/CoFeB/Pt in (**a**), respectively. Solid lines and dotted lines are the data measured with +H and −H, respectively.

2. Reconstruction of near field signals

We calculated the response function of 0.5-mm-thick ZnTe crystal in current experimental system, which can be used to reconstruct the near field signal of sample. The response function $\boldsymbol{H}_{spectr}(\omega)$ can be written as follows:

$$\boldsymbol{H}_{spectr}(\omega) = i\omega\chi^{(2)}I_0AL\left(1+\frac{1-n}{1+n}\right),$$

the ω is the angular frequency, the $\chi^{(2)} = (n+i\kappa)^2 - 1$ is the second order nonlinear coefficient of ZnTe crystal, the I_0 is the sampling laser pulse, the A is the attenuation coefficient, the L is coherent length of ZnTe, and the n is the refractive index of ZnTe in the THz spectral range. All parameters are the functions of angular frequency.

So, the near field signal in the frequency domain can be obtained by:

$$\boldsymbol{E}_{emit}(\omega) = \frac{\boldsymbol{E}_{EOS}(\omega)}{\boldsymbol{H}_{spectr}(\omega)},$$

the $\boldsymbol{E}_{EOS}(\omega)$ is the Fourier transformant of $\boldsymbol{E}_{EOS}(\mathrm{t})$, where the $\boldsymbol{E}_{EOS}(\mathrm{t})$ is the far field signal measured by experimentally. The time-domain near field of sample $\boldsymbol{E}_{emit}(t)$ can be obtained by inverse Fourier transformant of $\boldsymbol{E}_{emit}(\omega)$.

3. Current dynamics at different pump fluences

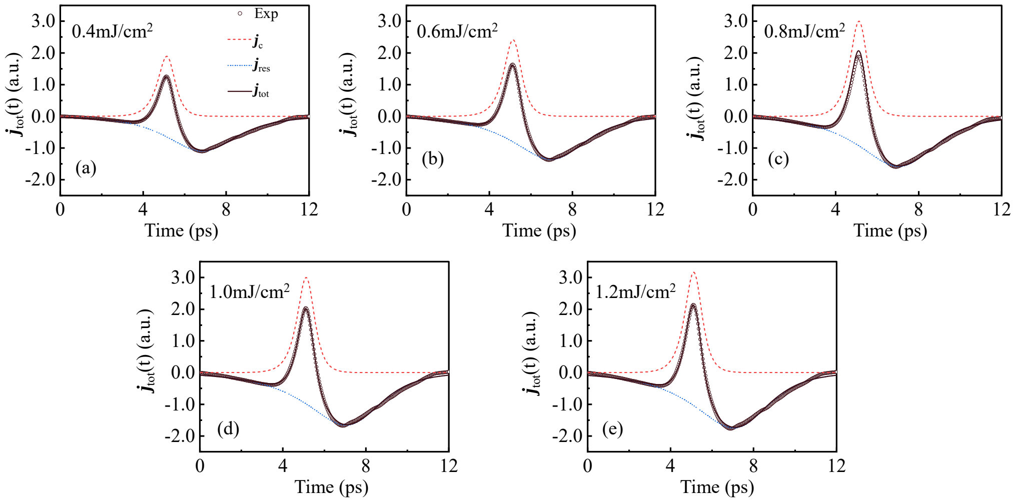

Figure S2. The fittings of charge currents with $\mathbf{j}_c(t)$ and $\mathbf{j}_{res}(t)$ contributions at difference pump fluence range from 0.4 mJ/cm^2 to 1.2 mJ/cm^2.
